# Editorial: Cross-Talk Between Inflammation and Barrier Framework at Mucosal Surfaces in the Lung: Implications for Infections and Pathology

**DOI:** 10.3389/fimmu.2020.598533

**Published:** 2020-09-29

**Authors:** Ramkumar Mathur, Gopal Murugaiyan, M. Nadeem Khan

**Affiliations:** ^1^Department of Geriatrics, School of Medicine and Health Sciences, University of North Dakota, Grand Forks, MD, United States; ^2^Ann Romney Center for Neurologic Diseases, Brigham and Women's Hospital and Harvard Medical School, Boston, MA, United States; ^3^Department of Biomedical Sciences, School of Medicine and Health Sciences, University of North Dakota, Grand Forks, ND, United States

**Keywords:** lung infection and pathology, mucosal inflammation, epithelial barrier function, mucosal immune response, therapeutic interventions

Inflammation at lung mucosal surfaces is required to resolve infections and preserve barrier integrity. A carefully orchestrated optimal immune response is a desirable outcome that leads to the resolution of infection while mounting a simultaneous reparative response to maintain the lung homeostasis and barrier integrity during acute and chronic inflammation. However, defects in immune regulation result in developing an aberrant immune response that causes immune pathology and exacerbates the disease. Therefore, the targets for immune therapies against acute and chronic inflammatory diseases in the lung require a comprehensive understanding of immune mechanisms associated with immune dysregulation and tissue damage. In this special issue, several original, review, and opinion articles highlighted the role of inflammation in acute infections and chronic conditions in the lung.

The perspective article from Alcorn encapsulates the significant role of interleukin−22 (IL-22) in promoting epithelial integrity and repair following the lung's infectious pathogen challenge. The pre-clinical animal models suggest that IL-22 has significant therapeutic potential in the context of infectious diseases. While the reparative role of IL-22 has been shown broadly in the context of epithelial repair and lung barrier integrity, the article highlights the need to further assess the effects of IL-22 on epithelial cells in inflammatory settings, perhaps in combination with pathogen-associated molecular patterns (PAMPs) or toxins.

The review article by LeMessurier et al. elaborates on the role of the respiratory barrier as a safeguard and regulator of defense against influenza and *Streptococcus pneumoniae*. The article highlights the role of leukocyte-epithelial as well as inter-epithelial crosstalk in the regulation of barrier integrity during influenza infection. Furthermore, the article elaborates the role of several host factors, such as TRAIL, interferons, and other inflammatory cytokines, in the altering epithelial junctions and permeability, which is associated with dysregulated inflammation, leading to the permissiveness of influenza-infected airway cells for *Streptococcus pneumoniae* co-infection. Finally, the article proposes that the crosstalk at the interface of microbial pathogens and human host epithelium presents multiple opportunities for the development of clinically relevant therapies during respiratory infections.

In a mini review article by Samarasinghe and Rosch, they described the convergence of inflammatory pathways in allergic asthma and sickle cell diseases (SCD). Asthma and SCD share a number of similarities in terms of the immunological factors associated with their respective disease states. The immunologic sequelae associated with SCD and asthma are complex but have some overlap. The review provided a concise overview of inflammatory pathways impacted during SCD and asthma, and how pulmonary physiology and inflammation are impacted during SCD and asthma comorbidity.

The original article by Allard et al. describes the role of asthmatic bronchial smooth muscle (BSM) derived CCL5 and its role in monocyte migration in response to the rhinovirus-infected epithelium. Asthma exacerbations, a significant concern in therapeutic strategies, are most commonly triggered by viral respiratory infections, particularly with human rhinovirus (HRV). The study assessed whether or not BSM could increase monocyte migration induced by HRV-infected bronchial epithelial cells. An *in vitro* model of co-culture of human bronchial epithelial cells in air-liquid interface with human BSM cells from control and asthmatic patients was developed to address that. HRV-induced monocyte migration was substantially increased in the co-culture model with asthmatic BSM, compared with control BSM. However, the well-known monocyte migration chemokine, CCL2, was not involved in this increased migration. Instead, the recruitment was CCL5 dependent. Therefore, the findings highlighted a new role of BSM cells in HRV-induced inflammation *via* CCL5.

Gao et al.'s original research article describes bacterial porin, OprC mediated impairment of host defense by increased quorum sensing mediated virulence of *Pseudomonas aeruginosa*. *P. aeruginosa*, found widely in the wild, causes infections in the lungs and several other organs in healthy people but more often in immunocompromised individuals. The authors reported that oprC deletion severely impaired bacterial motility and quorum-sensing systems, as well as lowered levels of lipopolysaccharide and pyocyanin in *P. aeruginosa*. In addition, oprC deficiency impeded the stimulation of TLR2 and TLR4 and inflammasome activation, resulting in decreased proinflammatory cytokines and improved disease phenotypes, such as attenuated bacterial loads, lowered lung barrier damage, and prolonged mouse survival. The findings summarize OprC as a critical virulence regulator, providing the groundwork for further dissection of the pathogenic mechanism of OprC as a potential therapeutic target of *P. aeruginosa*.

The original research article by Zhang et al. describes the Mycobacterium abscessus components and their crosstalk with human bronchial epithelial cells (HBECs). Mycobacterium avium complex (MAC) and Mycobacterium abscessus (MAB) are two of the most common causative pulmonary infection agents. The reaction between bronchial epithelia and components in the envelope of the mycobacterial cell wall is poorly understood. The results importantly demonstrate the role of Type I IFN in cross-talk between NHBE cells and MAB, suggesting an immune response by HBECs cells may play a central role in the imitation of innate immunity. Furthermore, the study underscores the importance of mycobacterial cell wall in initiating an innate immune response.

Finally, the review article by Aguilera and Lenz discusses the role of inflammation as a modulator of host susceptibility to influenza, pneumococcus, and co-infections. The article summarized the role of different leukocyte subsets and immune sensing to pulmonary infections. Specifically, the article elegantly summarizes the findings on alveolar macrophages, monocytes, NK cells, and cytokine mediators IFN-γ, TNF-α, IL-10 in influenza and influenza pneumococcal infections. The article concludes that regulation of lung innate immune responses in susceptible populations and in the context of complex environmental elements (such as the microbiota) are needed to provide avenues for the development of new treatments.

The contributions in the form of original and review articles to this Research Topic highlight the complex interplay between pathogen and inflammation in the lung and chronic conditions that dysregulate inflammation in a complex manner. The articles broadly underline the significance of safeguarding the mucosal barrier in the lung during infection or chronic inflammation by therapeutic interventions or tailoring immune response to allow more effective resolution of infection/inflammation and mitigate tissue damage and immune pathology.

## Author Contributions

RM, GM, and MK conceived, designed, and wrote the manuscript. All authors have read and approved the finalized version of the manuscript for publication.

## Conflict of Interest

The authors declare that the research was conducted in the absence of any commercial or financial relationships that could be construed as a potential conflict of interest.

